# A rare non-Robertsonian translocation involving chromosomes 15 and 21

**DOI:** 10.1590/1516-3180.2013.1316539

**Published:** 2013-12-01

**Authors:** Marcelo Razera Baruffi, Deise Helena Souza, Rosana Aparecida Bicudo Silva, Ester Silveira Ramos, Danilo Moretti-Ferreira

**Affiliations:** I BSc, PhD. Assistant Professor, Department of Genetics, Instituto de Biociências (IBB), Universidade Estadual Paulista (Unesp), Botucatu, São Paulo, Brazil.; II BSc, MSc. Biomedic, Department of Genetics, Instituto de Biociências (IBB), Universidade Estadual Paulista (Unesp), Botucatu, São Paulo, Brazil.; III BSc. Technician, Department of Genetics, Instituto de Biociências (IBB), Universidade Estadual Paulista (Unesp), Botucatu, São Paulo, Brazil.; IV MD, PhD. Assistant Professor, Department of Genetics, Faculdade de Medicina de Ribeirão Preto (FMRP), Universidade de São Paulo (USP), Ribeirão Preto, São Paulo, Brazil.; V BSc, PhD. Associate Professor, Department of Genetics, Instituto de Biociências (IBB), Universidade Estadual Paulista (Unesp), Botucatu, São Paulo, Brazil.

**Keywords:** Translocation, genetic, Down syndrome, Chromosomes, human, pair 15, Chromosomes, human, pair 21, Chromosome deletion, Translocação genética, Síndrome de Down, Cromossomos humanos par 15, Cromossomos humanos par 21, Deleção cromossômica

## Abstract

**CONTEXT:**

Robertsonian translocations (RT) are among the most common balanced structural rearrangements in humans and comprise complete chromatin fusion of the long arm of two acrocentric chromosomes. Nevertheless, non-Robertsonian translocation involving these chromosomes is a rare event.

**CASE REPORT:**

We report a *de novo* unbalanced translocation involving chromosomes 15 and 21. The newborn was the daughter of a 29-year-old mother and a 42-year-old father. The couple was non-consanguineous. Clinical findings led to the diagnosis of Down syndrome (DS) with severe congenital heart defects (persistent arterial duct, and complete atrioventricular septal defect), as well as low birth length and weight (< 5^th^ and < 10^th^ percentile, respectively, based on specific measurement curves for DS). Conventional cytogenetic analysis revealed the karyotype 46,XX,der(15)(15pter→15q26.2::21q11.2→21qter). The translocation was confirmed by means of fluorescence *in situ* hybridization. The parents had normal karyotypes.

**CONCLUSIONS:**

Differently from RT, in our case a rare event occurred involving the distal segment of 15q and the proximal segment of 21q. Only two reports of this translocation, involving chromosomes 15 and 21 but different breakpoints, have been described so far. The association between 21q duplication and 15q deletion makes it difficult to separate the effect of each chromosome, but might also be responsible for increasing the growth retardation, as detected in our case. Cytogenetic analysis on DS patients is mandatory not only to confirm the diagnosis, but also to assess the risk of recurrence at genetic counseling, as well as to evaluate the contribution of other chromosome aberrations in the final phenotype.

## Abstract (pt)

**CONTEXTO:**

Translocações robertsonianas (TR) estão entre os rearranjos estruturais balanceados mais comuns em humanos e compreendem a fusão da cromatina completa do braço longo de dois cromossomos acrocêntricos. No entanto, são raras as translocações não Robertsonianas envolvendo esses cromossomos.

**RELATO DE CASO:**

Nós descrevemos uma translocação não balanceada *de novo* envolvendo os cromossomos 15 e 21. A recém-nascida era filha de uma mãe de 29 anos e de um pai de 42 anos, casal não consanguíneo. Os achados clínicos levaram ao diagnóstico de síndrome de Down (SD) com defeitos cardíacos congênitos graves (persistência do canal arterial e defeito do septo atrioventricular completo), além de baixos comprimento e peso ao nascimento (< 5o e < 10o percentil em curvas de medidas específicas para SD, respectivamente). A análise citogenética convencional revelou o cariótipo 46,XX,der(15)(15pter→15q26.2::21q11.2→21qter). A translocação foi confirmada por hibridação *in situ* fluorescente. Os pais apresentavam cariótipo normal.

**CONCLUSÕES:**

Diferentemente das TR, nesse caso ocorreu evento raro envolvendo o segmento distal de 15q e o proximal de 21q. Apenas dois relatos dessa translocação, envolvendo os cromossomos 15 e 21 e diferentes pontos de quebra, já foram descritos. A associação entre duplicação 21q e deleção 15q dificulta a distinção dos efeitos de cada cromossomo, mas poderia também ser responsável pelo acentuado retardo de crescimento. A análise citogenética é obrigatória em pacientes com SD não apenas para confirmar o diagnóstico, mas também para avaliar o risco de recorrência no aconselhamento genético, bem como avaliar a contribuição de alterações de outros cromossomos no fenótipo final.

## INTRODUCTION

The majority (approximately 95%) of Down syndrome cases are caused by simple trisomy of chromosome 21. Translocations involving this chromosome account for approximately only 1-3% of all Down syndrome cases.^1^ ^-^ ^3^ Almost all of these translocations involve a Robertsonian translocation, which comprises the long-arm elements of two acrocentric chromosomes (chromosomes 13, 14, 15, 21 and 22).^3^

Less common distinct forms of Down syndrome result from structural changes in chromosome 21. For example, reciprocal translocations are extraordinarily rare and are the cause of less than 0.1% of Down syndrome cases. These translocations with partial trisomy have been used to define a Down syndrome critical region or Down syndrome *loci*, consisting of a segment of the long arm of chromosome 21.^1^ ^,^ ^2^

Recent studies have attempted to delineate an association between rare terminal deletions of the long arm of chromosome 15 and a specific phenotype, in particular short stature caused especially by the loss of one copy of the *IGF1R* (insulin-like growth factor receptor) gene.^4^ ^,^ ^5^

The main aim of this study was to report a very rare *de novo* non-Robertsonian translocation involving chromosomes 15 and 21, and to show the importance of cytogenetic investigation in all cases of clinical diagnosis of Down syndrome.

## CASE REPORT

A female newborn was referred to the Genetic Counseling Service of Universidade Estadual Paulista (Unesp), Botucatu, São Paulo, with a clinical diagnosis of Down syndrome. She was the only child of non-consanguineous parents. Her 29-year-old mother was normal and her 42-year-old healthy father had a normal daughter from a previous marriage. Ultrasound examination during pregnancy detected the presence of oligohydramnios, but no renal malformations. She was born at term by means of vaginal delivery with a birth length of 41 cm (< 5^th^ percentile), weight of 2000 g (< 10^th^ percentile) and head circumference of 33 cm (50^th^ percentile, all measurement based on a standard chart for Down syndrome).^6^

**Figure 1:**
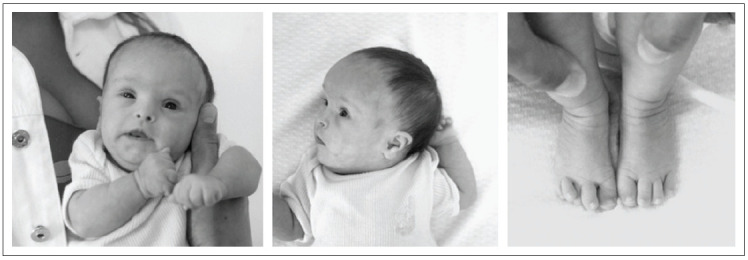
The patient at the age of three months. (A) Face, (B) profile, (C) feet (note the overlapping toes).

Clinical examination revealed hypotonia, brachycephaly, frontal bossing, flat face, upslanting palpebral fissures, protruding tongue, short neck with excess skin, brachydactyly, clinodactyly of the fifth fingers with a single interdigital crease on the right side and overlapping toes (Figure 1). At the age of three months, her length (51.5 cm) and weight (3,350 g) were still < 5^th^ and < 10^th^ percentiles, respectively, and her head circumference (36 cm) was normal, based on specific standard measurement charts for Down syndrome.^6^ Echocardiography showed a persistent arterial duct, and a complete atrioventricular septal defect, which included a ventricular septal defect.

Metaphase chromosome spreads were obtained from temporary lymphocyte cultures and the slides were subjected to Giemsa trypsin G-banding (GTG-banding). Fluorescence *in situ* hybridization (FISH) was performed using commercial probes. 

For chromosome 15, we used a digoxigenin-labeled chromosome 15 probe (Coatsome; Cat. No. P5216; ONCOR, Gaithersburg, MD, USA) and chromosome 15 α-satellite probe D15Z (Cat. No. P5033; ONCOR, Gaithersburg, MD, USA). Hybridization was performed in accordance with the manufacturer's instructions, followed by detection by means of fluorescein isothiocyanate (FITC)-labeled anti-digoxigenin and counting using propidium iodide staining (final concentration: 0.3 µg/ml in antifade).

For chromosome 21, we used the Chromoprobe Multiprobe System Octochrome (Cytocell). FISH was performed to investigate the centromere regions of chromosomes 15 and 21, using a Cytocell probe for the centromeres (α-satellites) of chromosome 13/21 (Cat. No. LPE 013G) and chromosome 15 (Cat. No. LPE 015G). Hybridization was carried out in accordance with the manufacturer's instructions.

**Figure 2:**
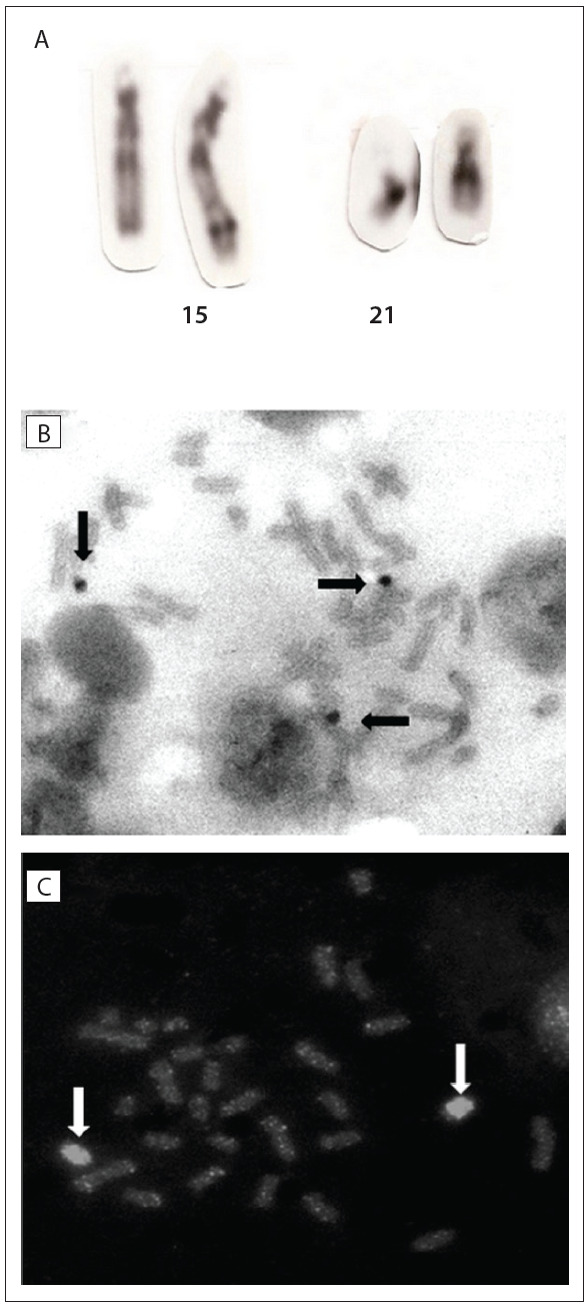
Cytogenetic analysis. (A) GTG-banded partial metaphase. Chromosomes are (from left to right): normal chromosome 15, der(15) and two normal chromosomes 21. (B) fluorescence hybridization in situ (FISH) using chromosome 21 probe. (C) FISH using chromosome 15 probe.

Cytogenetic analysis (Figure 2) revealed the karyotype 46,XX,der(15)(15pter→15q26.2::21q11.2→21qter). The parents presented normal karyotypes.

Unfortunately, the patient did not return for a follow-up and our service was unable to contact this specific patient/family due to a change in their address and phone number. Consequently, it was impossible to have more information about this case and to collect more blood samples.

## DISCUSSION

A translocation involving chromosome 21 has the potential to produce duplication of the Down syndrome critical region.^1^ ^,^ ^2^ Duplication of a long segment of 21q could explain the predominant Down syndrome phenotype, as detected in our case. Unlike in Down syndrome caused by trisomy of chromosome 21, terminal deletions of the long arm of chromosome 15 have rarely been described.^4^ ^,^ ^7^ ^-^ ^13^

A large number of genes have been mapped within the terminal 15q region, and among them, *IGF1R* seems to play the main role in the phenotype of 15qter syndrome. Copy number variations of this gene result in prenatal and postnatal growth restriction. Additionally, *IGF1R* contributes towards development of the central nervous and cardiovascular systems.^4^ ^,^ ^5^ ^,^ ^12^

Our study had some limitations due to the loss of contact with the patient. For example, we were unable to observe the IGF1 levels, which might have shown evidence of the IGF1R deficiency.

From reviewing the literature, we observed that the majority of the patients who were known to present terminal deletions of 15q displayed prenatal and postnatal growth retardation, cardiac defects, delayed development, ear abnormalities and clinodactyly.^4^ ^,^ ^5^ ^,^ ^7^ ^-^ ^13^ All these features and the Down syndrome findings overlap. For this reason, it was difficult to separate the effects of 21q duplication from those of 15q deletion, in our case. The association between these two chromosomal aberrations may have been responsible for increasing the growth retardation in our case. 

To the best of our knowledge (Table 1), only two reports on non-Robertsonian translocation, involving chromosomes 15 and 21, have been published so far. Abeliovich et al. described the karyotype t(15;21)(q15;q22.1)pat in two siblings.^14^ One of them had Prader-Willi syndrome. An interesting case of a patient with typical Down syndrome phenotype and apparently normal karyotype was studied by Nadal et al.^15^ Using FISH, these authors found the unbalanced karyotype t(15;21)(q26;q22.1).^14^ The father and other members of the family carried a balanced translocation between chromosomes 15 and 21. Although these cases have some similarities to our patient, these three translocations present different breakpoints. A deeper search with more complex and more expensive methods, such as array comparative genomic hybridization (CGH), might have clarified some points such as the precise breakpoints. Unfortunately, we were unable to obtain further blood samples from this patient.

**Table 1:** Results from our review of the medical databases (with and without using the words "case report" as a filter). Date of search: January 9, 2013

Database	Search strategy	Results
PubMed	Chromosome 21 AND Chromosome 15 AND Translocation AND Robertsonian	15*
	Chromosome 21 AND Deletion chromosome 15 AND translocation AND syndrome	06†
	Chromosome 21 AND deletion chromosome 15 AND translocation AND ‘Non Robertsonian'	01*
Embase	Chromosome and 15 and 21 and translocation and Robertsonian	214*
	Translocation non-Robertsonian	46*
	Translocation and non Robertsonian and acrocentric	02*
	15 AND chromosome AND 21 AND ‘Non-Robertsonian' AND Translocation	04*
Lilacs	Translocation AND chromosome 21	04*
	Translocation AND chromosome 15	05*
	Translocation AND chromosome 15	05*

* None of them involves chromosome 15 and 21 at the same time and/or Down syndrome; †Only one with a t(15;21).

On the other hand, the techniques carried out in our study were sufficient to show the rare non-Robertsonian translocation, the involvement of chromosomes 21 and 15 and the chromosome 21 trisomy. The results obtained provide evidence for the occurrence of this atypical chromosome aberration, and for the importance of cytogenetic analysis.

## CONCLUSION

An association between these two chromosomal aberrations could be responsible for increasing the growth retardation, as detected in our case.

Cytogenetic analysis on Down syndrome patients is mandatory, not only to confirm the diagnosis, but also to assess the risk of recurrence at genetic counseling, in particular when translocations are involved. Moreover, this makes it possible to evaluate the contribution of other chromosome aberrations to the final phenotype.
